# Impact of a hospital-wide computerised approach to optimise the quality of antimicrobial prescriptions in patients with severe obesity: a quasi-experimental study

**DOI:** 10.1186/s12879-021-06682-8

**Published:** 2021-09-18

**Authors:** Stéphanie Sirard, Vincent Nault, Marie-France Langlois, Julie Perron, Louis Valiquette

**Affiliations:** 1 Department of Microbiology and Infectious Diseases, Université de Sherbrooke, 3001, 12e Avenue Nord, Sherbrooke, Québec, J1H 5N4 Canada; 2 Medical Division, Lumed Inc., Sherbrooke, Québec, J1H 5C7 Canada; 3 Department of Medicine, Division of Endocrinology, Université de Sherbrooke, Québec, J1H 5N4 Canada; 4 Centre de Recherche du Centre Hospitalier Universitaire de Sherbrooke, Québec, J1H 5N4 Canada

**Keywords:** Obesity, Antimicrobial, Prescription, Antimicrobial stewardship

## Abstract

**Background:**

Rates of adherence to available recommendations for dose adjustments in patients with severe obesity are generally low. Hence, antimicrobials are often underdosed in these patients. Antimicrobial stewardship programmes can improve the use of antimicrobials in hospitalised patients. The aim of the study was to analyse the impact of an antimicrobial stewardship programme based on a computerised clinical decision support system for optimal dosing and antimicrobial use in inpatients with severe obesity.

**Methods:**

This quasi-experimental retrospective study using interrupted time series was conducted in an academic centre in Canada from August 2008 to June 2018. The Antimicrobial Prescription Surveillance System was implemented in August 2010 (intervention 1) and specific rules targeting patients with class III obesity (body mass index ≥ 40 kg/m^2^) were added in June 2014 (intervention 2). Data were collected from all hospitalised adults receiving antimicrobials which required dose adjustment for severe obesity and were stratified by body mass index. Segmented regression analysis of interrupted time series was used to evaluate the impact of the Antimicrobial Prescription Surveillance System on the proportion of inappropriate days of therapy according to posology and on antimicrobial consumption.

**Results:**

Overall, 65 205 antimicrobial prescriptions (68% non-obese, 25% class I-II obesity, and 7% class III obesity) were analysed. In patients with class III obesity, the intervention was associated with a decrease in the proportion of inappropriate days of therapy (trend after the first intervention, −0.8% per 2-month period [95% CI −1.1 to −0.5], *p* < 0.001; intercept, 11.3% [95% CI 8.2 to 14.5], *p* < 0.001), which led to a reduction of 35% over an eight-year period (from pre-intervention level of 19.1%). Intervention 1 resulted in a downward trend in antimicrobial consumption, followed by an increasing trend after intervention 2. In these patients, the most frequent interventions made by pharmacists targeted posology (46%).

**Conclusions:**

Antimicrobial Prescription Surveillance System had a positive impact on dosing optimisation and antimicrobial consumption in patients with class III obesity. Improving antimicrobial prescriptions in these patients is important because suboptimal dosing could be associated with unfavourable outcomes.

**Supplementary Information:**

The online version contains supplementary material available at 10.1186/s12879-021-06682-8.

## Background

Over the last decades, the prevalence of obesity among adults has increased steadily. In 2016, it was estimated at 13.1% globally, reaching almost 30% in some countries [1].Obesity not only increases the risk of infection [2–4] but also causes physiological changes, altering the pharmacokinetics (PK) of several antimicrobials, in both critically ill and non-critically ill patients [5–7]. Underdosing antimicrobials in patients with obesity can lead to sub-inhibitory concentrations, which in turn can decrease treatment efficacy [8, 9]. In contrast, supratherapeutic doses may lead to toxicity in patients with obesity [10, 11].

Despite guidelines suggesting adjustments and PK/PD data, physicians do not necessarily adjust antimicrobials in patients with class III obesity and, when local recommendations are implemented, they are not necessarily followed by prescriptors [12–16]. In the entire hospitalised population, 30–50% of antibiotic use is inappropriate [17–19]. Patients with class III obesity are more likely to receive inadequate dosing than patients with other body mass index (BMI) [12, 13, 15, 16, 20].

Implementing antimicrobial stewardship programmes (ASPs) using prospective audit and feedback has shown consistent positive impacts on antimicrobial prescription [21–23]. Only a few interventions have been described in patients with obesity, but have led to interesting results, such as improvement of the adherence rates to guidelines [24], increase in dose adjustments [25, 26], and reduction in the rates of dose errors [27] and costs [28].

Since 2010, the Antimicrobial Prescription Surveillance System (APSS), a computerised decision support system (CDSS) designed to support prospective audit and feedback interventions, has been used by the ASP team in our centre. It has been associated with significant and sustained reductions in length of stay (LOS) in patients receiving antimicrobials, along with antimicrobial consumption and costs [23]. This system includes dose adjustment rules for special populations, such as patients with severe obesity.

This study aimed to evaluate the impact of an ASP using APSS on inappropriate antimicrobial dosing, antimicrobial use, and hospital LOS in patients with class III obesity.

## Methods

### Population and study design

This quasi-experimental, retrospective study was conducted at the Centre intégré universitaire de santé et de services sociaux de l’Estrie-Centre hospitalier universitaire de Sherbrooke (CIUSSSE-CHUS), a 677-bed academic centre in the Province of Quebec, Canada. Approval was obtained from the CIUSSSE-CHUS institutional ethics review board (# 12–187).

All adults (≥ 18 years) with documented weight and height values and at least one prescription for IV or oral antimicrobial, hospitalised between 18 August 2008 and 17 June 2018, were included. All antimicrobial prescriptions were assessed, regardless of the number per patient. Prescriptions of antimicrobials requiring no adjustment for obesity (Additional file 1: Table S1) were excluded. Patients hospitalised in the maternity and psychiatry wards were excluded because BMI measurement is inaccurate in parturient women and because these populations were not targeted by the ASP team.

### Data collection

Data were retrospectively extracted from a clinical data warehouse (Centre Informatisé de Recherche Évaluative en Services et Soins de Santé, CIRESSS) and APSS (Lumed Inc., Sherbrooke, Canada), both used at the CIUSSSE-CHUS. Prescription data (dose and dosing intervals, route of administration, and length of treatment) and data on hospitalised patients (age, sex, BMI) were collected. Patients were grouped according to their BMI: non-obese (< 30 kg/m^2^), class I–II obesity (30–39.9 kg/m^2^), and class III obesity (severe obesity) (≥ 40 kg/m^2^). Any available height value and the last available weight value within 12 months of admission were taken.

### Intervention

APSS is a CDSS that allows post-prescription review by generating alerts for potentially inappropriate antimicrobial prescriptions based on locally approved guidelines. APSS monitors relevant clinical information and identifies deviations from optimal treatment in posology, duration of treatment, route of administration, drug interactions, and drug-bug mismatches. Based on published data and local experts, special rules were developed for dose adjustment in patients with class III obesity (Additional file 1: Table S2).

The intervention (intervention 1) was previously described elsewhere [23]. Briefly, APSS was implemented on 18 August 2010. During the first year of implementation, a clinical pharmacist was assigned to the APSS on weekdays for 15 h a week. Then, it increased to 30 h a week. During the first intervention, the dosing adjustments in patients with class III obesity were left to the discretion of the antimicrobial stewardship pharmacists because the knowledge base was not specialised for this population. As a consequence, they were performed mainly on patients with severe infections as they had prolonged antimicrobial treatments. In June 2014, specific rules targeting patients with a BMI ≥ 40 were added to APSS (intervention 2) but the application of the dosage modification remained at the discretion of the pharmacists and the prescribers. During the study period, prospective audit and feedback triggered by APSS were the only stewardship activities conducted at the CIUSSSE-CHUS.

There is no computerized physician order entry system in place for prescribing at the CIUSSSE-CHUS. Prescriptions are written by physicians and then entered into the CIUSSSE-CHUS electronic health record system by technical assistants at the pharmacy department, creating a lag of one to two hours for revision by pharmacists. Only therapeutic prescriptions are analysed by APSS, no alerts are produced on prophylactic agents.

### Outcomes

The primary outcome was the proportion of inappropriate days of therapy (DOT) (number of inappropriate DOT/total number of DOTs). Other outcomes of interest were antimicrobial consumption in DOT per 1000 inpatient days (PD) and defined daily doses per 1000 inpatient days (DDD/1000 PD), and average LOS in patients receiving antimicrobials. DOT were deemed inappropriate if dose or dosing intervals did not match our guidelines. Outcomes were compared before (pre-intervention: August 2008 to August 2010) and after the implementation of APSS (interventions 1 and 2), stratified by BMI classes. One DOT represents the administration of any amount of a specific antimicrobial on a given day [29], and DDD corresponds to the assumed average maintenance dose per day for a drug used for its main indication in adults [30].

### Statistical analysis

Differences among BMI groups were determined using the χ^2^ test with a *p* value of < 0.05. We used segmented regression analysis of interrupted time series [31] to evaluate the impact of APSS on the proportion of inappropriate days of therapy and on antimicrobial consumption. This method allowed visual graphic analysis and assessment of the changes in the level and trend over time between pre-intervention (August 2008 to August 2010) and post-intervention (intervention 1: August 2010 to June 2014; intervention 2: June 2014 to June 2018). The level change is a measurement of the sudden change between what the model would have predicted without the intervention at the period of the intervention and the value obtained without the intervention on the first period where the intervention was on.

Our data set comprised 59 periods: 12 periods before and 47 following the intervention. Data were combined into periods of two months to ensure that the numbers were sufficient to use the segmented regression analysis. A stepwise approach was used to model the time series, and the stationarity of outcomes was evaluated with a Dickey-Fuller test (5% level). The predictions were graphically plotted against the observations, and the residuals plotted against a normal distribution (normality was tested using the Jarque–Bera test to validate whether they were randomly distributed). The Durbin-Watson test was used to detect autocorrelation in residuals, and a value close to 2 was chosen to indicate non-autocorrelation. The most parsimonious model was identified using bilateral significance tests. Data were analysed using the PROC AUTOREG statement in SAS version 9.4 (SAS Institute, Cary, NC) with a lag order of 6 to consider annual seasonality.

## Results

Overall, 74 676 patients were hospitalised during the study period, accounting for 79 031 hospitalisations (17% with antimicrobials which require a dosing adjustment for obesity). A total of 72 796 antimicrobial prescriptions were identified during the study period, among which a patient’s BMI value was available for 65 205 (90%). Included prescriptions were divided according to the following categories: non-obese (68%), class I–II obesity (25%), and class III obesity (7%). The distribution of patients’ sex (49% males) and the upward trend in age (45% of patients aged 65 and over) remained stable over the study period.

The most prescribed antimicrobials in patients with class III obesity were piperacillin-tazobactam (25%), ciprofloxacin (18%), and cefazolin (9%) (Table 1). The same proportions were observed in non-obese and class I–II obesity patients.

**Table 1:** Most prescribed antimicrobials and their inadequacy rates regarding dosing adjustment in patients with class III obesity

	Total number of prescriptions (%)	Total number of inadequate prescriptions (%)
Piperacillin-tazobactam	1332 (25.3)	184 (13.8)
Ciprofloxacin	930 (17.6)	60 (6.4)
Cefazolin	489 (9.3)	227 (46.4)
Amoxicillin-clavulanate	426 (8.1)	66 (15.3)
Metronidazole	257 (4.9)	97 (37.7)
Vancomycin	248 (4.7)	68 (27.4)
Ceftriaxone	213 (4.0)	13 (6.1)
Meropenem	186 (3.5)	43 (23.1)
Cephalexin	165 (3.1)	46 (27.8)
Imipenem	163 (3.1)	27 (16.6)
Penicillin	108 (2.0)	2 (1.9)
Clindamycin	107 (2.0)	63 (58.9)
Total^a^	5271	1051

^a^Antimicrobials prescribed with a frequency of < 2% (less than 100 prescriptions): azithromycin (n = 95), levofloxacin (n = 84), ampicillin (n = 82), amoxicillin (n = 77), fluconazole (n = 73), gentamicin (n = 50). Other antimicrobials with 50 prescriptions or less are not reported

The ratio of prescriptions for IV and PO antimicrobials (59:41) was relatively similar over the study period, but the proportion of prescriptions for IV antimicrobials was significantly higher in the class III obesity group than in the other groups (61.3 vs 59.2, *p* = 0.008).

### Inappropriate days of therapy

In all antimicrobials with at least 100 prescriptions, the most inappropriately prescribed agents according to posology were clindamycin (58.9%), cefazolin (46.4%), and metronidazole (37.7%) (Table 1). Overall, prescriptions were the most inappropriate in patients with class III obesity compared to other patients (19.9% vs 6.1%, *p* < 0.001).

**Fig. 1:**
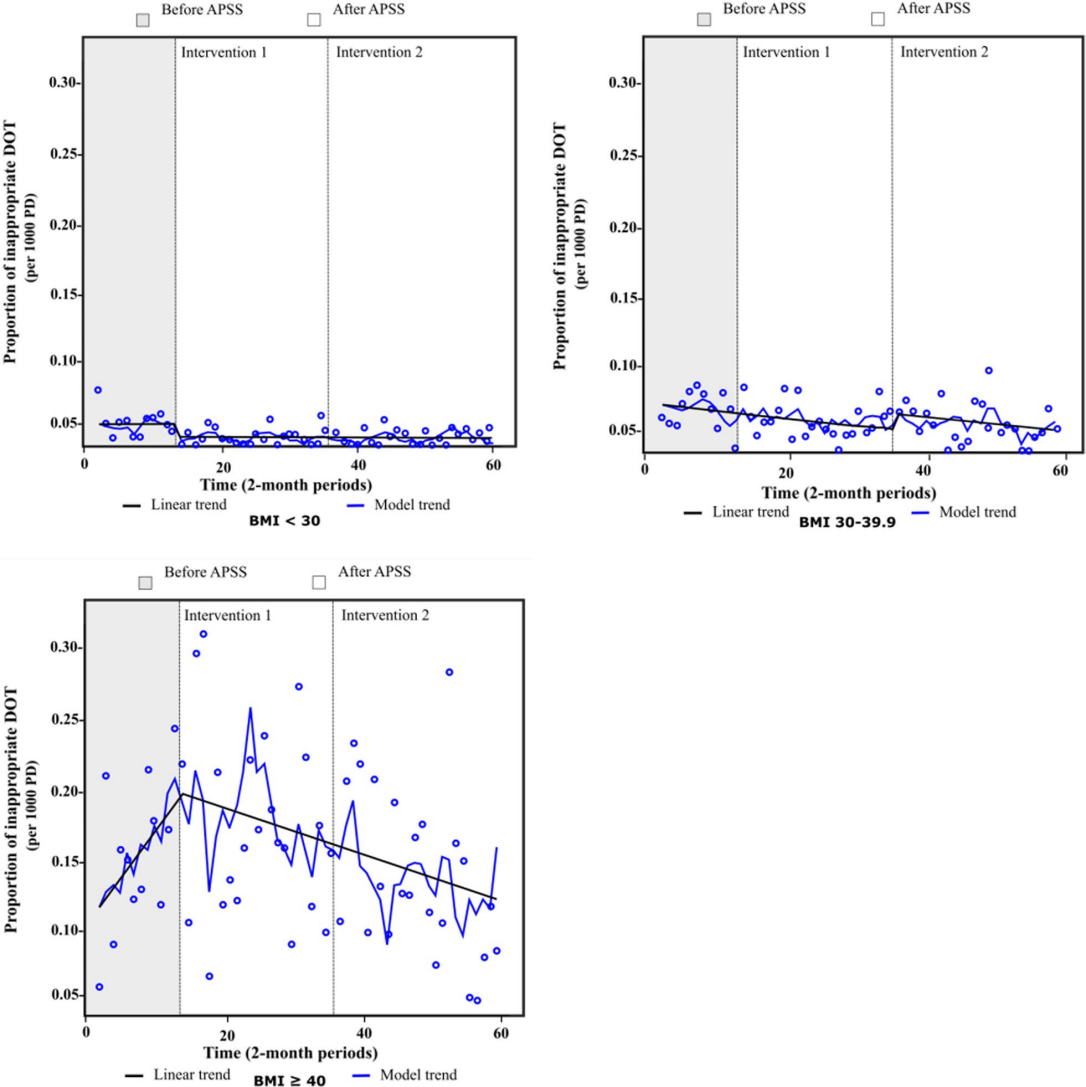
Proportion of inappropriate DOT per 1000 PD for antimicrobials requiring an adjustment for obesity. Total number of inappropriate DOT per 1000 PD divided by the total number of DOT, by 2-month periods

Figure 1 illustrates the impact of APSS on the proportion of inappropriate DOT per 1000 PD for antimicrobials requiring an adjustment for severe obesity in the three groups of patients. The effect of intervention 1 was mostly observed in non-obese patients with an immediate reduction of 24% in inappropriate DOT, indicated by a change in the level (−1.1% [95% CI −1.5 to −0.7], *p* < 0.001; intercept 4.6% [95% CI 4.4 to 4.9], *p* < 0.001) (Table 2). In patients with class I–II obesity, the proportion of inappropriate DOT increased immediately after intervention 2, but was close to statistical significance (1.3% [95% CI −0.03 to 2.6], *p* = 0.056; intercept 7.0% [95% CI 6.2 to 7.8], *p* < 0.001). For patients with class III obesity, from a base level of 11.3% ([95% CI 8.2 to 14.5], *p* < 0.001) of inappropriate DOT and a significant upward trend (0.7% [95% CI 0.4 to 0.9], *p* < 0.001), intervention 1 showed a significant change in the trend. This impact was not instantaneous, but gradual, and was sustained over time (−0.8% [95% CI −1.1 to −0.5], *p* < 0.001). Compared to the pre-intervention level (19.1% of inappropriate DOT), interventions 1 and 2 combined led to a 35% reduction in inappropriate DOT at the end of the study.

**Table 2:** Changes in outcomes after interventions 1 and 2 using segmented regression analysis (2008–2018)

BMI	Outcomes	Intercept at time zero (95% CI)	*p*	Trend (95% CI)	*p*	Level change after Int1^a^ (95% CI)	*p*	Trend change after Int1^b^ (95% CI)	*p*	Level change after Int2^a^ (95% CI)	*p*	Trend change after Int2^b^ (95% CI)	*p*
< 30	Proportion of inappropriate DOT (%)	4.6 (4.4 to 4.9)	< 0.001	−0.0004 (−0.01 to 0.01)	0.9	−1.1 (−1.5 to −0.7)	< 0.001	NS		NS		NS	
	DOT per 1000 PD	436.1 (381.0 to 491.2)	< 0.001	14.9 (7.2 to 22.6)	< 0.001	-78.7 (-137.9 to -19.5)	0.01	−16.1 (−23.7 to −8.4)	< 0.001	NS		NS	
	DDD per 1000 PD	360.7 (313.7 to 407.7)	< 0.001	4.1 (−0.3 to 8.5)	0.07	NS		−6.2 (−11.1 to −1.2)	0.02	NS		NS	
	LOS (days)	8.0 (7.6 to 8.3)	< 0.001	0.03 (0.02 to 0.04)	< 0.001	NS		NS		NS		NS	
30–39.9	Proportion of inappropriate DOT (%)	7.0 (6.2 to 7.8)	< 0.001	−0.06 (−0.1 to −0.02)	0.003	NS		NS		1.3 (−0.03 to 2.6)	0.056	NS	
	DOT per 1000 PD	364.1 (328.8 to 399.4)	< 0.001	16.4 (11.6 to 21.3)	< 0.001	−88.0 (−126.8 to −49.1)	< 0.001	−16.6 (−21.5 to -11.7)	< 0.001	−96.1 (−126.3 to −65.9)	< 0.001	NS	
	DDD per 1000 PD	236.3 (202.1 to 270.6)	< 0.001	19.7 (15.0 to 24.5)	< 0.001	−107.5 (−147.9 to −67.1)	< 0.001	−22.1 (−26.8 to −17.4)	< 0.001	−78.6 (−106.1 to −51.1)	< 0.001	2.8 (1.0 to 4.6)	0.004
	LOS (days)	8.5 (7.7 to 9.4)	< 0.001	−0.01 (−0.05 to 0.03)	0.5	NS		NS		3.6 (2.3 to 4.9)	< 0.001	−0.1 (−0.2 to −0.07)	< 0.001
≥ 40	Proportion of inappropriate DOT (%)	11.3 (8.2 to 14.5)	< 0.001	0.7 (0.4 to 0.9)	< 0.001	NS		−0.8 (−1.1 to −0.5)	< 0.001	NS		NS	
	DOT per 1000 PD	615.0 (569.4 to 660.6)	< 0.001	−7.1 (−10.5 to −3.7)	< 0.001	114.9 (24.6 to 205.3)	0.01	NS		NS		7.9 (1.7 to 14.2)	0.01
	DDD per 1000 PD	496.7 (456.8 to 536.6)	< 0.001	−9.9 (−12.9 to −6.9)	< 0.001	180.0 (100.3 to 259.7)	< 0.001	NS		NS		12.6 (7.1 to 18.0)	< 0.001
	LOS (days)	11.5 (9.9 to 13.1)	< 0.001	0.1 (0.04 to 0.2)	0.001	−4.7 (−7.3 to −2.1)	0.001	NS		NS		NS	

CI, confidence interval; DDD, defined daily dose; DOT, days of therapy; Int1, intervention 1; Int2, intervention 2; LOS, length of stay; NS, not statistically significant; PD, inpatient day

^a^Measure of immediate impact of the intervention; ^b^Measure of long-term impact of the intervention

### Antimicrobial consumption

**Fig. 2:**
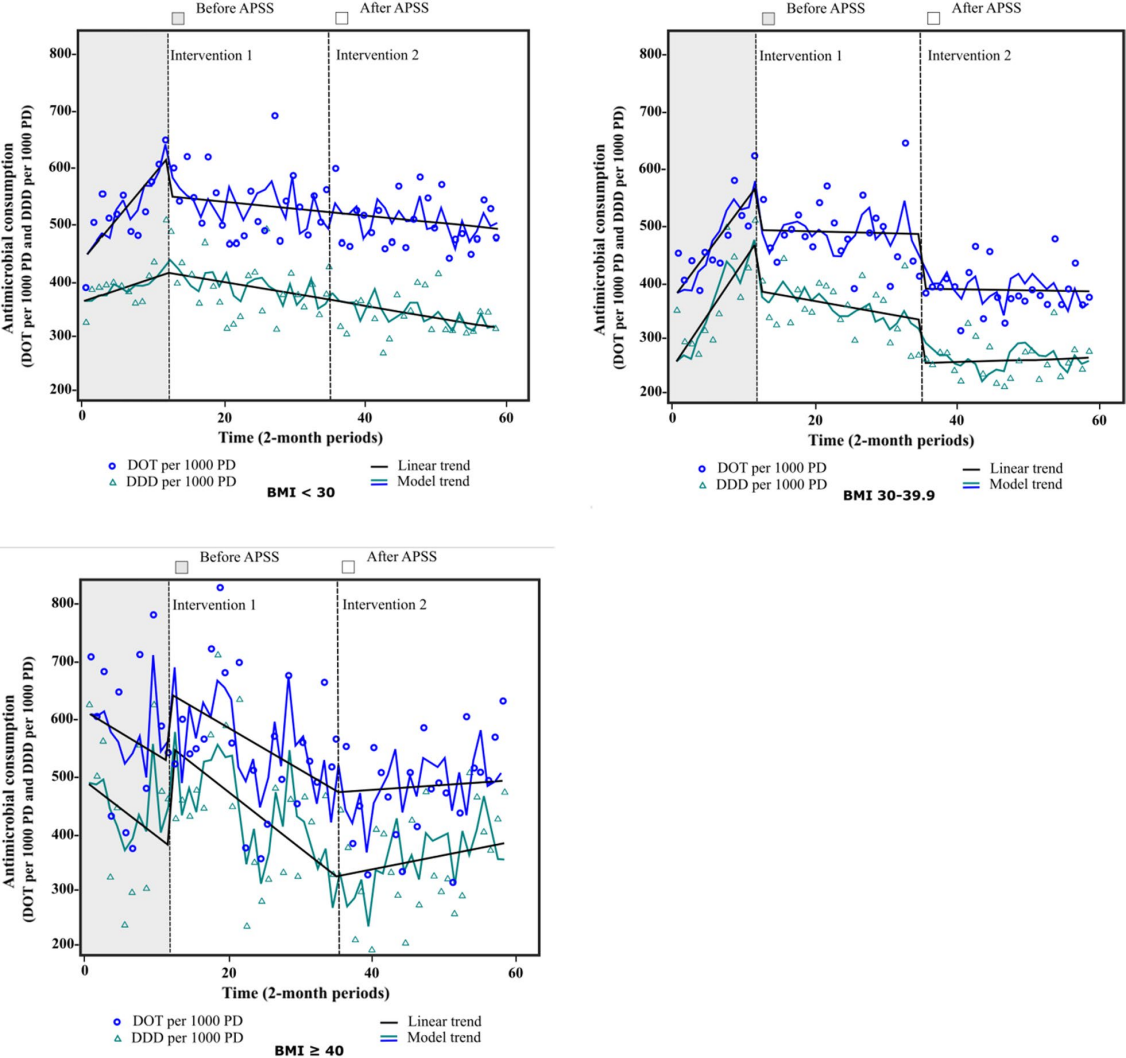
Antimicrobial consumption in DOT and DDD (per 1000 PD)

Figure 2 illustrates the impact of APSS on antimicrobial consumption in DOT and DDD (per 1000 PD) for selected antimicrobials. In patients with class III obesity, from a pre-intervention level of 529.9 DOT per 1000 PD, although there was a downward trend following intervention 1, it was not significant (Table 2). A similar pattern was observed with DDD, with a higher rising trend after intervention 2 (12.6 [95% CI 7.1 to 18.0], *p* < 0.001 vs 7.9 [95% CI 1.7 to 14.2], *p* = 0.01). In non-obese patients, following intervention 1, there was an immediate impact, indicated by a change in the level (−78.7 [95% CI −137.9 to −19.5], *p* = 0.01; intercept 436.1 DOT per 1000 PD [95% CI 381.0 to 491.2], *p* < 0.001), along with a sustained impact, indicated by a change in the trend of DOT (−16.1 [95% CI −23.7 to −8.4], *p* < 0.001). A sustained reduction was also observed in DDD following intervention 1 in these patients (−6.2 [95% CI −11.1 to −1.2], *p* = 0.02). In patients with class I-II obesity, from a pre-intervention level of 561.2 DOT per 1000 PD and a significant upward trend, intervention 1 had an immediate (−88.0 [95% CI −126.8 to −49.1], *p* < 0.001; intercept 364.1 DOT per 1000 PD [95% CI 328.8 to 399.4], *p* < 0.001) and long-term impact (−16.6 [95% CI −21.5 to −11.7], *p* < 0.001), which was accentuated with intervention 2 (−96.1 [95% CI −126.3 to −65.9], *p* < 0.001). The reduction was higher in DDD, with an immediate impact of 45% following intervention 1. The impact was also significant over time, followed by an immediate effect of intervention 2. We observed a reduction of 18% and 24% in antimicrobial consumption (DOT) in non-obese and class I–II obesity patients, respectively.

### Average LOS

The average LOS in patients receiving selected antimicrobials was relatively stable over the study period. Although similar in non-obese and class I–II obesity patients, the average LOS with antimicrobials was higher in patients with class III obesity (Additional file 1: Fig. S1). Across all groups, the mean LOS was lower in hospitalisations without antimicrobials. In patients with class III obesity, intervention 1 had an immediate impact (−4.7 days [95% CI −7.3 to −2.1], *p* = 0.001; intercept 11.5 days [95% CI 9.9 to 13.1], *p* < 0.001), but was not maintained over time as the upward trend continued (Table 2). In non-obese patients, none of the interventions had an effect on LOS, and the upward trend initiated before APSS was not reversed. In patients with class I-II obesity, LOS immediately increased after intervention 2 (3.6 days [95% CI 2.3 to 4.9], *p* < 0.001), but then gradually decreased over time (−0.1 days [95% CI −0.2 to −0.07], *p* < 0.001) and returned to a level similar to pre-intervention 2.

### Recommendations

Overall, 9343 recommendations were reported during the study period in included patients (non-obese: 67%, class I–II obesity: 22%, class III obesity: 11%) for selected antimicrobials. The rate of recommendations per hospitalisation with selected antimicrobials was higher in patients with class III obesity than in other patients (1.27 vs 0.68, *p* < 0.001). The most frequent interventions made by pharmacists in patients with class III obesity concerned posology (n = 455, 46%), switch from intravenous to oral therapy (n = 105, 11%), and discontinuation of treatment (n = 79, 8%) (Table 3). They were consistent across all BMI groups, except that intervention for posology was significantly more frequent in the class III obesity group than in the other groups (46% vs 29%, *p* < 0.001). The acceptance rate by physicians was 93.9% and was similar to the rate noted in the other groups (92.3% in class I–II obesity vs 92.7% in non-obese).

**Table 3:** Recommendations in patients with class III obesity

	No. approved	No. rejected	Total	Acceptation rate (%)	Frequency (%)
Posology	424	24	455	93.2	46.1
Switch from IV to oral therapy	91	12	105	86.7	10.6
Discontinuation of treatment	78	1	79	98.7	8.0
Duration	70	1	71	98.6	7.2
Microbiology	66	3	70	94.3	7.1
Missing dosage target	39	0	39	100	4.0
Interaction	38	0	38	100	3.9
Off target serum concentration	23	0	23	100	2.3
Restricted antimicrobials	21	0	21	100	2.1
Other^a^	77	7	86	90.0	8.7
Total	927	48	987^b^	93.9	100

^a^Less than 2%: cost (n = 18), individualized intervention (n = 12), renal toxicity (n = 10), laboratory test request (n = 9), prolonged infusion (n = 9), antimicrobial associated with *C. difficile* risk (n = 6), spectrum redundancy (n = 6), allergy (n = 4), post hemodialysis dose (n = 4), asymptomatic bacteriuria (n = 3), exception (n = 3), antiviral after negative testing (n = 2)

^b^Unknown acceptance status in 12 interventions

## Discussion

With the increasing prevalence of obesity worldwide and because individuals with obesity are likely to be treated with more complex antimicrobial treatments [32], increased attention to this population using ASPs is needed. Patients with obesity are frequently underdosed [13, 15, 16, 20], and dose adjustments are necessary for several antimicrobials [7]. Over an eight-year period, the implementation of a CDSS in our centre had a positive impact on the concordance between the prescriptions of antimicrobials and local dosing guidelines in patients with class III obesity, but also an impact on antimicrobial consumption in non-obese and class I-II obesity patients.

In our study, the inadequacy rates of prescriptions according to posology in patients with class III obesity exceeded 38% for some agents. This finding is consistent with that of several studies that have reported low adherence to dosing guidelines for frequently used antimicrobials in this population [12, 13, 15, 16, 20, 33]. We classified prescriptions as inadequate based only on their doses and dosing intervals. The observed concordance would have been even lower if other types of errors would have been taken into account (e.g. presence of criteria for a switch to oral treatment).

We observed different patterns in the three groups of patients following both interventions in the proportions of inappropriate days of therapy. The effect of APSS was the most marked in patients with class III obesity, who are at higher risk of underdosing and complications. The highest proportion of inappropriate DOT was reported in patients with class III obesity, for whom there was an upward trend before the implementation of APSS. Although interventions were not performed systematically in patients with class III obesity until 2014, intervention 1 reversed this trend and had a gradual impact that was maintained over 8 years. The reduction in the proportion of inappropriate DOT was accentuated with intervention 2 due to more systematic interventions based on the development of local guidelines for patients with class III obesity. Without the second intervention, a plateau might have been observed, instead of a downward trend.

We reported a positive impact of APSS on antimicrobial consumption in all three weight groups. In addition to the inclusion of specific alerts for underdosing in class III obesity, intervention 2 was based on an improved version of APSS with several additional rules and algorithms targeting new populations of patients, but also a more performant graphical user interface facilitating clinical review of each case. In non-obese patients, a reduction in DOT per 1000 PD occurred after intervention 1 only (level change), but after both interventions in patients with class I-II obesity. In patients with class III obesity, there was a downward trend following intervention 1. Then, DOT per 1000 PD increased with intervention 2 and the introduction of specific rules. The return to a rise was observed in all models of antimicrobial consumption. A pattern similar to DOT per 1000 PD was reported when measuring DDD per 1000 PD. However, a greater rising trend was observed in DDD compared with DOT following intervention 2, while it is difficult to explain the increase in the average duration of treatment (increase in DOT), the increase in DDD reflects the increase in the doses targeted by our intervention.

No significant impact was observed in LOS in patients receiving selected antimicrobials, as it remained relatively stable during the study period. However, we observed great variance, especially in patients with class III obesity. Given the low number of patients with class III obesity, the sickest patients with long LOS significantly affected the data. Compared with other patients, they generally have longer LOS because of several comorbidities and higher risks of complications and infections [34–37]. Moreover, patients with obesity receive more prescriptions of several drugs, including antimicrobials [38] and receive more antimicrobials [39] than patients without obesity. They are also more likely to receive complex antimicrobial therapy, namely intravenous antimicrobials and longer courses of therapy compared with other patients [32]. We found a 2% difference in the proportion of prescriptions for IV antimicrobials between the class III obesity group and the other groups, but it may not be clinically relevant. Physicians may be reluctant to switch to the oral route for fear that the treatment will be less effective. Severe obesity was also associated with greater alternate level of care LOS (delayed discharge without need for active care) [40]. All these factors may explain the greater DOT and longer LOS in patients with obesity.

The high acceptance rate to our recommendations in class III obesity shows the beliefs of the risk of underdosing in this population by the physicians in our centre. We could perform a high number of recommendations because of the automated detection process included in APSS. Pharmacists’ interventions were influenced by the severity of the infection, the degree of obesity and the prior response to non-adjusted treatment (33).

Only a few studies have reported strategies intended to optimise antimicrobial prescriptions in patients with obesity. A surgical prophylaxis policy including an automatic substitution for higher doses led to a greater proportion of patients with BMI ≥ 30 receiving higher doses of cefazolin and vancomycin (15% versus 63% and then 72% following the introduction of a preoperative pause) [25]. The implementation of a pharmacy dose optimisation programme increased the compliance rates to institutional guidelines (74.8% versus 45.1%, *p* < 0.001) in patients weighing ≥ 80 kg [24]. These studies included limited numbers of patients with obesity [25] or used different definitions of obesity and were limited to surgical prophylaxis patients.

A pharmacist-led institutional protocol that identifies patients with obesity and set automatic dose adjustments for seven antimicrobials was associated with a dose adjustment in 40% of cases and a compliance rate to the protocol of 89% [26]. Golik Mahoney and Adra [41] showed that the use of a computerised prescriber order entry with decision support decreased the proportion of patients with obesity who had received supratherapeutic doses of acyclovir (100%, 10/10 to 46%, 6/13). An electronic dose calculator led to a reduction in gentamicin dose errors (43% to 20%) and in interval error rates (12.8% to 4.0%) in patients with a weight > 20% above ideal body weight (IBW), two years after its implementation [27]. Once again, these studies were limited by a small number of patients with obesity and evaluated only a few different antimicrobials. Overall, the impact of these interventions was assessed over a relatively short period of time. In the emergency department, other interventions such as computerised provider order entry systems [20] and the presence of a pharmacist [42] have improved the dosing of several antimicrobials. Other authors have presented ASP including rules or criteria for patients with a weight > 100 kg [43, 44]. However, the impact of these strategies on antimicrobial prescriptions has not been reported.

One of the primary limitations of this study is that it reports results from a single centre. The CIUSSSE-CHUS is a teaching hospital, and medical residents are present in most departments, thus making response to feedback easier because one member of the team is available most of the time. Generalisation to hospitals with different realities may not be possible. Another limitation is the absence of randomisation. Even if quasi-experimental studies are the strongest non-experimentation studies because they allow for seasonality (especially if taking into account two years before and eight years after), confounders may have influenced the impact of the intervention. The absence of a control group limits the possibilities of attributing positive effects to the intervention. Thus, this study design does not allow to establish the causality between the use of APSS and the outcomes studied, but rather to identify associations. Prescriptions are reviewed on weekdays only, but prescriptions on weekends may have only a small impact on our results. This study was also limited by the low number of patients with class III obesity hospitalised in our centre, making some analyses impossible to perform because of a great variance. In the same way, we could not analyse data by antimicrobial or even by class, because of insufficient numbers of prescriptions of some agents (e.g. 74 prescriptions of aminoglycosides during the study period). Serum concentrations of antimicrobials were not measured, so it was impossible to accurately assess the appropriateness of the patients’ regimens, other than by relying on the concordance of the prescription to our guidelines. Moreover, antimicrobial dosing in obesity still requires additional research and there is uncertainty about optimal dosing for many antimicrobials, especially in patients with class III obesity. Hence guidelines are not necessarily informed by high quality data and this may impact adherence to recommendations.

To our knowledge, this is the first study to assess the sustained impact of a computerised decision support system led by an ASP team in patients with class III obesity. We have previously shown a three-fold increase in the median appropriateness of the treatment of bloodstream infections and a reduction in inadequate prescriptions upon discharge [33]. Moreover, we could compare the impact of APSS according to the weight of patients and BMI was available in 90% of hospitalised patients in our centre.

## Conclusions

In conclusion, we showed the positive impact of an antimicrobial stewardship programme on the adequacy of prescriptions along with antimicrobial consumption, especially in patients with class III obesity in our centre. Considering the high frequency of underdosing and a large proportion of IV antimicrobials, interventions targeting these patients have the biggest potential impact. Dosing adjustment is recommended for most antimicrobials, but the impact of such adjustments on clinical outcomes remains unclear in patients with class III obesity. The impact of a CDSS on outcomes related to antimicrobial treatment such as the rate of readmission, the rate of relapse for infection and overall mortality could be interesting to study, but as already highlighted in a prior study, several methodological challenges must be overcome to study these outcomes [33]. Besides antimicrobials, a CDSS could improve prescriptions for other drug classes likely to be inadequate in patients with class III obesity (such as anticoagulants, anaesthetics), but this remains to be investigated.

## Supplementary Information

**Additional file 1: Table S1.** Recommendations for antimicrobial dosing adjustment in patients with class III obesity at the CIUSSSE-CHUS. **Table S2**. Dosing regimens for the most prescribed antimicrobials for which an adjustment has been considered in patients with class III obesity at the CIUSSSE-CHUS. **Figure S1.** Average length of stay in patients with selected antimicrobials.

## Data Availability

The datasets used and analysed during the current study are available from the corresponding author on reasonable request. Other data are included in Additional file 1.

## Abbreviations

ASP: Antimicrobial stewardship programme
APSS: Antimicrobial Prescription Surveillance System
BMI: Body mass index
CDSS: Computerised decision support system
CI: Confidence interval
CIUSSSE-CHUS: Centre intégré universitaire de santé et de services sociaux de l’Estrie-Centre hospitalier universitaire de Sherbrooke
DDD: Defined daily dose
DOT: Day of therapy
LOS: Length of stay
PD: Inpatient day
PK: Pharmacokinetics
