# Supplementary material for: Impact of a hospital-wide computerised approach to optimise the quality of antimicrobial prescriptions in patients with severe obesity: a quasi-experimental study
Source: BMC Infect Dis. 2021 Sep 18;21:972. doi: 10.1186/s12879-021-06682-8 (PMC8449866; doi:10.1186/s12879-021-06682-8)
Supplement: Supplementary file 1 — Additional file 1: Table S1. Recommendations for antimicrobial dosing adjustment in patients with class III obesity at the CIUSSSE-CHUS. Table S2. Dosing regimens for the most prescribed antimicrobials for which an adjustment has been considered in patients with class III obesity at the CIUSSSE-CHUS. Figure S1. Average length of stay in patients with selected antimicrobials. [file 12879_2021_6682_MOESM1_ESM.docx]

**Supplementary data**

**Table S1.** Recommendations for antimicrobial dosing adjustment in patients with class III obesity at the CIUSSSE-CHUS

| **Antimicrobials for which an adjustment has been considered (increase in dose)** | Amoxicillin, amoxicillin-clavulanate, ampicillin, aztreonam, azithromycin, cefaclor, cefadroxil, cefazolin, cefepime, cefotaxime, cefoxitin, cefprozil, ceftazidime, ceftolozane/tazobactam, ceftriaxone, cefuroxime, cephalexin, ciprofloxacin, clindamycin, fluconazole,  imipenem/cilastatin, levofloxacin, linezolid, meropenem, metronidazole, penicillin, piperacillin/tazobactam, ticarcillin-clavulanate |
| --- | --- |
| **Details and rules about the calculation of the doses of some antimicrobials** | - acyclovir : use of adjusted body weight if actual body weight is > 130% ideal body weight (often a downward adjustment) - amikacin, gentamicin, tobramycin : use of adjusted body weight if actual body weight is > 130 % ideal body weight (often a downward adjustment) - amphotericin B : use of ideal body weight or lean body weight if BMI > 35 (often a downward adjustment) - colistimethate : use lower of ideal or actual body weight - daptomycin : use of adjusted body weight if BMI > 30 - ganciclovir : use of adjusted body weight if BMI > 40 - linezolid : adjustment needed if body weight is > 150 kg with normal renal function - trimethoprim/sulfamethoxazole high dose therapy: use of adjusted body weight if actual body weight is > 130 % ideal body weight (often a downward adjustment) - vancomycin : loading dose based on actual body weight and renal function, then based on PK calculations - voriconazole : use of adjusted body weight if BMI > 30, then based on serum levels |
| **Antimicrobials for which an adjustment has not been considered** | Caspofungin, cefixime, clarithromycin, cloxacillin, doxycycline, ertapenem, itraconazole, micafungin, moxifloxacin, nitrofurantoin, oseltamivir, posaconazole, rifampicin, tigecycline |

**Table S2**. Dosing regimens for the most prescribed antimicrobials for which an adjustment has been considered in patients with class III obesity at the CIUSSSE-CHUS

| **Creatinine Clearance *** | | | | |
| --- | --- | --- | --- | --- |
| **Antimicrobial** | **>50 mL/min** | **30–50 mL/min** | **10–30 mL/min** | **< 10 mL/min** |
| **Penicillins** |  |  |  |  |
| piperacillin/  tazobactam | (CrCl >40 mL/min) 3.375g q4h or 4.5g q6h | (CrCl 20–40 mL/min) 3.375g q6h | (CrCl 0–20 mL/min) 2.25g q6h | (CrCl 0–20 mL/min) 2.25g q6h |
| amoxicillin-clavulanate (PO) | (CrCl >30 mL/min) 875 mg q8h | (CrCl >30 mL/min) 875 mg q8h | 500 mg q8h | 500 mg q12h |
| amoxicillin | (CrCl >30 mL/min) 1g q6-8h | (CrCl >30 mL/min) 1g q6-8h | 500 mg q8h | 500 mg q12h |
| **Cephalosporins** |  |  |  |  |
| cefazolin | 2000 mg q4-6h | (CrCl 35–50mL/min) 2000 mg q8h | (CrCl 10–35 mL/min)  2000 mg q12h | 2000 mg q24h |
| ceftriaxone | 2000 mg q12h | 2000 mg q12h | 2000 mg q12h | 2000 mg q12h |
| **Quinolones** |  |  |  |  |
| ciprofloxacin  (IV) | 400 mg q8-12h | 400 mg q12h | 400 mg q24h | 400 mg q24h |
| (PO) | 500-750 mg q12h | 500 mg q12h | 500 mg q24h | 500 mg q24h |
| **Others** |  |  |  |  |
| metronidazole  (IV/PO) | 7.5 mg/kg q6h (maximum daily dose: 4g) | 7.5 mg/kg q6h (maximum daily dose: 4g) | 7.5 mg/kg q6h (maximum daily dose: 4g) | 3.75 mg/kg q6h |
| vancomycin^a^  (IV) | (CrCl >60 mL/min) 15 mg/kg q8h + loading dose | (CrCl 30–60mL/min) 15 mg/kg q24h + loading dose | 15 mg/kg q48h + loading dose | 10-15 mg/kg q72h + loading dose |
| (PO) | 125 mg q6h | 125 mg q6h | 125 mg q6h | 125 mg q6h |

Abbreviations: CrCl: creatinine clearance, IV: intravenous, PO: oral administration.

* estimated with the Cockcroft-Gault equation with adjusted body weight

^a^ Suggested doses are initial doses and vary depending on the pathogen and the severity of the infection

**Figure S1.** Average length of stay in patients with selected antimicrobials

**
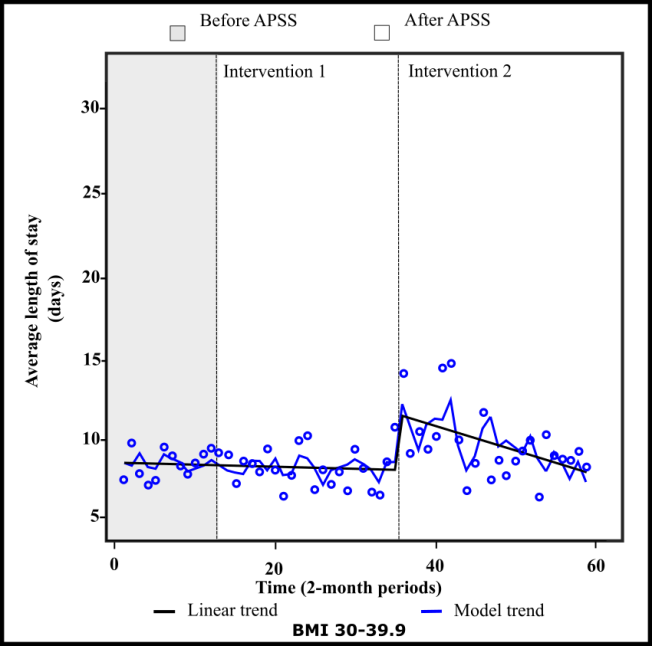

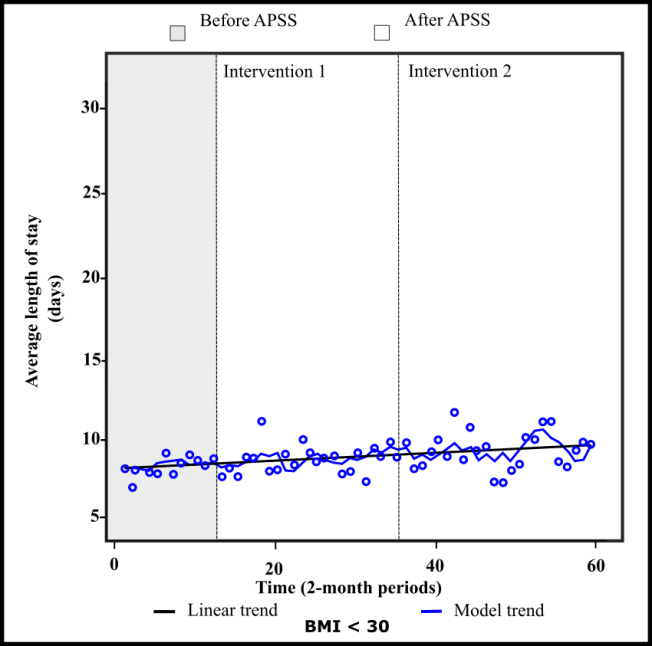
**

**
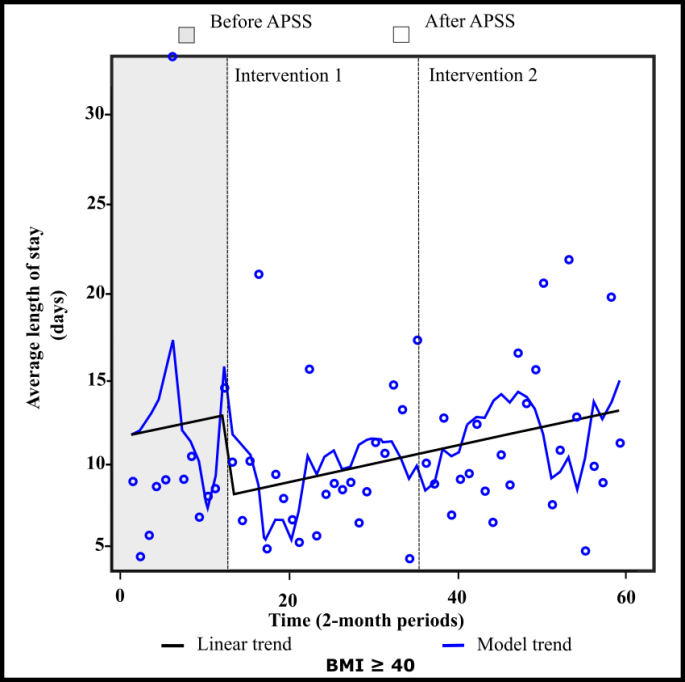
**
